# Whither Are We Drifting?

**Published:** 1895-12

**Authors:** W. C. Barrett

**Affiliations:** Buffalo


### Whither are We Drifting?

BY W. C. BARRETT, M.D., D.D.S., BUFFALO.

Abstract of paper read at American Dental Association, Aug., 1895.

Dr. Barrett in his paper showed how, from the time of the Revolutionary war, when there was an average of one hundred and seventy-five thousand people for each dentist, there had been a proportionate increase of both population and dentists, until in 1895 it averaged one dentist for every two thousand six hundred of population. "Yet," he said, "the amount of work done by each individual dentist is very much greater than when there was but one practitioner for each 175,000 of the population. * * *

"It is true that dentists have greatly multiplied in number, and we constantly hear the wails of pessimists who declare that the colleges are turning out graduates at a rate that must soon make their number more than that of their patients. The prophesies are usually heard from old-time practitioners, who have not broadened their practice with the advance of modern ideas, or kept pace with the progress of professional events. To them the greater part of the dentistry of to-day has no existence, for their horizon has not been pushed back during the last five decades. With their restricted conception of dental practice, it is no wonder that they cannot see where room shall be found for the constantly augmenting number that come thronging through the gates, and they condemn the colleges in no gentle terms for launching such hordes of practitioners into a stream that, to their conception, now seems absolutely turbid with them. They forget that these same colleges are year by year digging that stream deeper and making it wider in a much greater ratio than they are peopling it with occupants."

Further, the writer inquired what should be done to get more dentists into our American Dental Associations. He cited the fact that the country was so large that each year some would have to go so far to reach the place of meeting that they staid away. There was an inconstant attendance, in the majority, and therefore acquaintances were not so thoroughly formed. As a remedy he said it would seem to him that we naturally tend toward four aggregations: one for the east, one for the west and northwest, one for the south and southwest, and one for the trans-montane portions of our common country.

